# DHEA Protects Human Cholangiocytes and Hepatocytes against Apoptosis and Oxidative Stress

**DOI:** 10.3390/cells11061038

**Published:** 2022-03-18

**Authors:** Ewa Kilanczyk, Dagmara Ruminkiewicz, Jesus M. Banales, Piotr Milkiewicz, Małgorzata Milkiewicz

**Affiliations:** 1 Department of Medical Biology, Pomeranian Medical University, 70-204 Szczecin, Poland; d.ruminkiewicz@gmail.com (D.R.); milkiewm@pum.edu.pl (M.M.); 2 Department of Liver and Gastrointestinal Diseases, Biodonostia Health Research Institute, Donostia University Hospital, University of the Basque Country (UPV/EHU), CIBERehd, Ikerbasque, 20014 San Sebastian, Spain; jesus.banales@biodonostia.org; 3 Department of Biochemistry and Genetics, School of Science, University of Navarra, 31009 Pamplona, Spain; 4 Translational Medicine Group, Pomeranian Medical University, 70-204 Szczecin, Poland; p.milkiewicz@wp.pl; 5 Liver and Internal Medicine Unit, Medical University of Warsaw, 02-097 Warsaw, Poland

**Keywords:** DHEA, cholangiocytes, apoptosis

## Abstract

Primary biliary cholangitis (PBC) is a rare chronic cholestatic and immune-mediated liver disease of unknown aetiology that targets intrahepatic bile duct cells (cholangiocytes) and primarily affects postmenopausal women, when their estrogen levels sharply decrease. An impaired cholangiocyte response to estrogen characterizes the terminal stage of the disease, as this is when an inefficiency of cholangiocyte proliferation, in balancing the loss of intrahepatic bile ducts, is observed. Here, we report that the estrogen precursor dehydroepiandrosterone (DHEA) and its sulfate metabolites, DHEA-S and 17 β-estradiol, enhance the proliferation of cholangiocytes and hepatocytes in vitro. Flow cytometry analysis showed that DHEA and DHEA-S decreased glyco-chenodeoxycholic acid (GCDC)-driven apoptosis in cholangiocytes. Cell viability assay (MTT) indicated that ER-α, -β, and the G-protein-coupled estrogen receptor, are involved in the protection of DHEA against oxidative stress in cholangiocytes. Finally, immunoblot analysis showed an elevated level of steroid sulfatase and a reduced level of sulfotransferase 1E1 enzymes, involved in the desulfation/sulfation process of estrogens in cirrhotic PBC, and primary sclerosis cholangitis (PSC) liver tissues, another type of chronic cholestatic and immune-mediated liver disease. Taken together, these results suggest that DHEA can prevent the deleterious effects of certain potentially toxic bile acids and reactive oxygen species, delaying the onset of liver disease.

## 1. Introduction

Cholangiocytes are epithelial cells that line the bile ducts and are preferentially damaged in primary biliary cholangitis (PBC), a chronic cholestasis liver disease with autoimmune phenomena. The accumulation of bile acids, which is characteristic of cholestasis, leads to the destruction of intrahepatic bile ducts, a condition termed ductopenia. In the early stages of the disease, the progression of ductopenia may be delayed by an enhanced proliferation of cholangiocytes [1]. It has been suggested that sex hormone dysfunction may contribute to PBC, as it mostly affects women during the postmenopausal period, when their estrogens levels decrease. Estrogens regulate growth, differentiation, and the metabolism of different cells and tissues that express estrogen receptors. Cholangiocytes express the alpha and beta estrogen receptors (ER-α and β). While the expression of ER-β is stable and elevated during all PBC stages, the expression of ER-α positively correlates with disease progression [2]. The G-protein-coupled estrogen receptor (GPER) also regulates estrogen action in the liver; this is a membrane receptor that binds to 17β-estradiol (E2) [3] and dehydroepiandrosterone (DHEA) [4], with high affinity.

Women who receive long-term estrogen replacement therapy may develop liver diseases [5]. However, in contrast, it has also been suggested that this therapy has no effect on disease development [6]. Estrogens are used in rodents as an experimental model of intrahepatic cholestasis [7,8,9] and their administration has, therefore, been avoided in patients with PBC. Clinical and experimental studies have suggested that estrogens modulate cholangiocyte survival [10,11] and play a role in PBC pathophysiology. The actions of estrogens are tightly regulated by enzymes, such as sulfotransferase (SULT) 1E1, which catalyzes estrogen sulfation/deactivation [12,13]. Aside from the regulation of hormone activities, sulfation catalyzes the detoxification of xenobiotics and bile acids [14].

DHEA (3β-hydroxy-5-androstene-17-one) and its 3β-sulfated metabolite, DHEA-S, are endogenous, circulating steroid hormones, produced in the adrenal glands and gonads. Plasma levels of DHEA and DHEA-S decline with age [15], and DHEA-S levels have consistently been reported to be lower in patients with chronic liver diseases, such as PBC [16]. In premenopausal women, 40–75% of the estrogens are derived from the peripheral metabolism of DHEA-S, whereas in postmenopausal women, this percentage is over 90% [17]. DHEA is a crucial precursor of steroid hormones, as it is metabolized to active androgens, including 5-dihydrotestosterone (DHT), in the adrenal glands, liver, and peripheral tissues. Androgens are metabolized to 17 β-estradiol (E2) or estrone (E1) by aromatase (CYP19). In addition, DHEA binds ER-α and ER-β with a binding affinity of Kd 1.2 and 0.5 μM, respectively, and androgen receptors with a Kd of 1.1 μM. DHEA also binds a number of hepatic nuclear receptors, such as peroxisome proliferator-activated receptor alpha (PPARα), as well as pregnane X receptor (PXR) and androstan receptor (CAR), which regulates the transcription of CYP genes [18]. Moreover, DHEA binds and activates a DHEA-specific membrane estrogen receptor, G-protein-coupled estrogen receptor (GPER), with a Kd of 49 pM [19]. The regeneration of active DHEA occurs in tissues via the action of steroid sulfatase (STS). This enzyme plays a pivotal role in regulating the formation of biologically active steroids, as it catalyzes the conversion of DHEA-S to DHEA. Interestingly, among postmenopausal women, the major source of estradiol is adrenal DHEA-S, which is converted to estrogens in fat tissue via STS [15]. This may indicate that the actions of this enzyme are important in the production of estrogens in postmenopausal women, especially in those who suffer from PBC.

Given that the enhanced proliferation and survival of cholangiocytes could delay the progression of ductopenia, the aim of this study was to examine the effect of the estrogen precursor DHEA and its metabolites (DHEA-S, E2, DHT, adione, and adiol) on their proliferation and protection against apoptosis, induced by toxic bile acid (GCDC) in: (i) normal cholangiocytes (i.e., normal human cholangiocytes (NHC) primary culture or SV-40 immortalized human cholangiocytes (H69)), (ii) PBC-like cholangiocytes with overexpression of miR-506 (H69-miR506) [20], and (iii) hepatocytes (Hep-G2 cells). We also evaluated the involvement of estrogen and androgen receptors in the apoptosis induced by GCDC and oxidative stress induced by tert-butylohydrochinon (tBHQ). In addition, the expression of SULT1E1 and STS in cirrhotic PBC liver tissue was examined.

## 2. Materials and Methods

### 2.1. Cell Culture and Tissue Preparation

Human cholangiocytes (NHC, H69, and H69-miR506) were cultured as previously described [21]. H69-miR506 cells were used as a model of PBC. It was previously reported that PBC cholangiocytes are characterized by miR-506 overexpression, which is involved in the direct targeting of the chloride/bicarbonate exchanger AE2 and the InsP3R3, leading to cholestatic and immune-activating features mimicking PBC [20,22]. In this regard, H69-miR506 cells are characterized by (i) elevated level of PDC-E2 (the main autoantigen of PBC); (ii) enhanced oxidative stress; (iii) increased level of pro-inflammatory interleukins (Il17 and IL23) [20]. Human hepatocytes (Hep-G2) were purchased from American Type Culture Collection (Manassas, VA, USA). Hep-G2 cells were grown in Eagle’s Minimum Essential Medium containing 10% fetal bovine serum (FBS; Gibco, Waltham, MA, USA), 100 U/mL penicillin, and 100 µg/mL streptomycin (Sigma-Aldrich, St Louis, MO, USA). Cultures were maintained in the presence of 5% CO_2_ at 37 °C.

Control liver tissues (*n* = 12) were obtained from large-margin liver resections of colorectal metastases. Liver tissues with histologically verified cirrhosis were collected during liver transplantations in PBC (*n* = 10) or PSC (another type of chronic cholestatic and immune-mediated liver disease; *n* = 10) patients. Please see the baseline clinical characteristics of PBC and PSC patients in Table 1. Total lysate from the control and cirrhotic liver tissues (PBC and PSC) was prepared as previously described [23].

### 2.2. MTT Assay

To assess the involvement of estrogen and androgen receptors in DHEA’s protection against oxidative stress induced by tBHQ, an MTT cell viability assay (3-(4,5-dimethylthiazol-2-yl)-2,5-diphenyltetrazolium bromide; Thermo Fisher, Waltham, MA, USA) was performed (see Supplementary Figure S1). To accomplish this, cells (NHC, H69, H69-miR506 and Hep-G2) were cultured in 96-well plates (10,000 cells/well). After 24 h, the cells were incubated with the following estrogen or androgen inhibitors: 12 nM of G15 (GPER), 2 nM of ICI 182,780 (ER-α), 10 nM of PHTTP (ER-β) and 2 nM of bicalutamide (androgen receptor). Two hours later, the cells were incubated with tBHQ together with DHEA. Please see the time-line scheme of experiments (Supplementary Figure S2). Cell viability was examined by measuring the reduction in yellow, water-soluble tetrazolium salt to purple, water-insoluble formazan. Data are presented as the percentage of survival relative to control conditions.

### 2.3. Cell Proliferation and Apoptosis

**Scheme 1:**
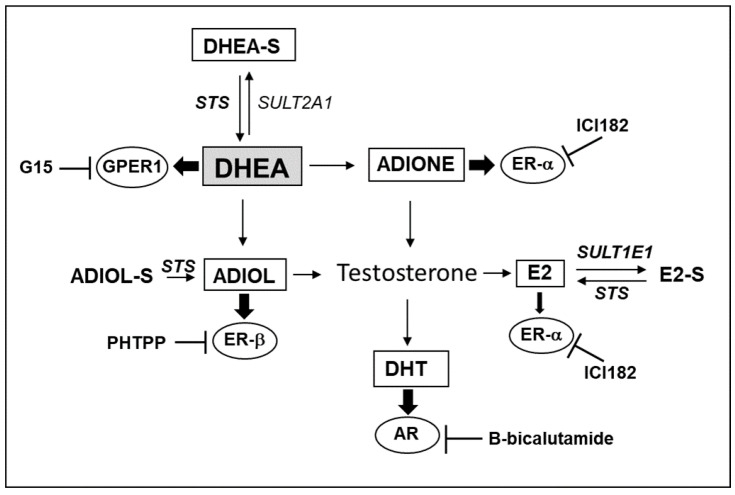
Metabolic conversion of DHEA [24]. DHEA and its metabolites used in experiments are in square frames. AR—androgen receptor; B-bicalutamide—androgen receptor inhibitor; DHT—dihydrotestosterone; Adiol—androstenediol; Adione—androstenedione, ER—estrogen receptor; E2—17-β estradiol; G15—G-coupled estrogen receptor 1 (GPER1) inhibitor; ICI182—estrogen receptor α inhibitor; PHTPP—estrogen receptor β inhibitor; STS—steroid sulfatase; SULT2A1—sulfotransferase 2A1; SULT1E1—estrogen sulfotransferase.

To measure the proliferation of cholangiocytes (NHC, H69, H69-miR506) and the hepatocytes (Hep-G2), the cells were incubated with a 1 nM DHEA, DHEA-S, E2 and adiol or 1 ng/mL of adione and DHT (Scheme 1). After 48 h, the cells were harvested from the culture flasks and the number of cells was determined using a CytoFLEX LX flow cytometer (Becton Dickinson, Franklin Lakes, NJ, USA). The acquisition time was 60 s, and the sample flow rate was set at 60 μL/minute.

Flow cytometric measurements (Annexin V/FITC and PI double staining) were used to quantify the extent of apoptosis in the total cell population. Cells were incubated for 24 h with 1 nM of DHEA, DHEA-S, E2, adiol or 1 ng/mL of adione and DHT, followed by pre-incubation (2 h with GCDC).

To evaluate the role of estrogen receptors in GCDC-driven apoptosis, cells were incubated with the following estrogen or androgen inhibitors: 12 nM of G15 (GPER), 2 nM of ICI 182,780 (ER-α), 10 nM of PHTTP (ER-β), and 2 nM of bicalutamide (androgen receptor). After 24 h, the cells were centrifuged and then resuspended in binding buffer to obtain concentrations of 100 cells/mL. Next, 5 µL FITC Annexin V and 5 µL PI were added to the sample tubes. Cells were softly vortexed and then incubated for 15 min in the dark at room temperature. Staining with 400 μL of binding buffer was added to each tube and the samples were analyzed using CytoFLEX LX flow cytometry (Beckman Coulter, Indianapolis, IN, USA) and CytExpert (Beckman Coulter) data analysis software. Each analysis was preceded by generating a quality control device report.

### 2.4. SDS-PAGE and Immunoblotting

Proteins (40 μg) extracted from control and cirrhotic liver tissues were electrophoresed on sodium dodecyl sulfate (SDS) polyacrylamide gels. Proteins were then blotted onto PVDF membranes (Thermo Scientific, Waltham, MA, USA) under semi-dry transfer conditions. Then membranes were incubated in a blocking buffer consisting of 5% non-fat dry milk in TBS-T for 1 h at room temperature. The primary antibodies anti-SULT1E1 (Sigma, St. Louise, MO, USA, #HPA028728) and anti-STS (Abcam, Cambridge, UK, #62219) were used at a 1:500 dilution, and anti-GAPDH (Cell Signaling, Danvers, MA, USA, #9139) was used at a 1:3000 dilution and incubated overnight at 4 °C. Afterwards, membranes were washed and incubated for 2 h at room temperature with anti-rabbit (Amersham ECL, Amersham, UK, #NA9340) or anti-mouse (Jackson ImmunoResearch, Philadelphia, PA, USA, #115-035-146) HRP-conjugated secondary antibodies at a 1:5000 dilution in TBS-T with 5% non-fat milk. After washing, membranes were incubated with enhanced chemiluminescence detection reagents (Millipore) and visualized and quantified with the MicroChemi 2.0 System and GelQuant software (DNR Bio-Imaging Systems, Neve Yamin, Israel). Densitometric analysis was performed in relation to GAPDH.

### 2.5. Immunohistochemical Analysis

The localization of SULT1E1 and STS proteins was examined in frozen liver sections. Briefly, frozen sections (6 μm) were fixed with acetone at −20 °C for 5 min. Then, the endogenous activity of peroxidase was blocked by treating the sections with 3% hydrogen peroxide for 10 min followed by exposure to an Avidin/Biotin blocking kit (Vector Laboratories, Burlingame, CA, USA, #SP-2001). The nonspecific-binding sites were blocked by incubating with 10% of normal horse serum in PBS for 30 min at RT. Following this step the sections were incubated with the primary antibodies: anti-SULT1E1 (Sigma, #028728, diluted 1:250) and anti-STS (Abcam, #62219, diluted 1:100) for 24 h at 4 °C. Then, the tissue samples were rinsed and incubated with a biotinylated anti-mouse/anti-rabbit IgG (#BA-1400, Vector Laboratories, Burlingame, CA, USA ) secondary antibodies for 60 min at RT. The reactions were visualized using ABC Vectastain and DAB kits (Dako, Agilent Technologies, Hovedstaden, Denmark). Then, the sections were counterstained with Mayer’s hematoxylin, dehydrated, and mounted. The negative control, in which the primary antibodies were omitted, was included in the study and uniformly demonstrated no reaction. Images were acquired with a ZEISS Axio Imager Z2 fluorescence microscope (Zeiss, Oberkochen, Germany).

### 2.6. Statistical Analysis

All data were analyzed using StatView software version 5 (SAS Institute Inc., Carry, NC, USA). Significant differences were determined using Fisher’s PLSD test. A value of *p* < 0.05 was considered significant.

## 3. Results

### 3.1. The Effect of DHEA and Its Metabolites on Cholangiocyte and Hepatocyte Proliferation and Apoptosis

**Figure 1:**
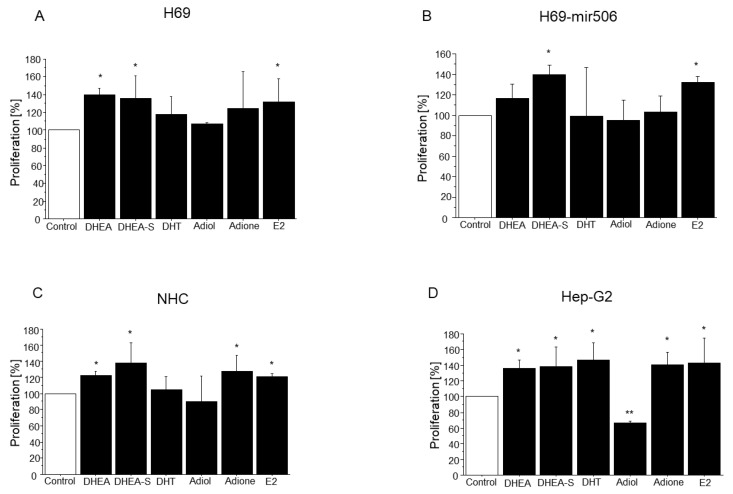
The effect of DHEA and its metabolites on the proliferation of cholangiocytes (H69, H69-miR506, and NHC) and hepatocytes (Hep-G2). Different cells types i.e., cholangiocytes (**A**–**C**) and hepatocytes (**D**) were incubated with DHEA and its metabolites (DHEA-S, DHT, adiol, adione and E2). Cells were harvested 48 h after treatment by scraping and were counted using CytoFLEX LX flow cytometer. Acquisition time was 60 s, and the sample flow rate was set at 60 μL/min. Results are presented as a mean ± SEM (*n* = 3); * *p* < 0.05; ** *p* < 0.01.

As cholangiocytes proliferation is relevant to PBC progression, we tested whether the estrogen precursor DHEA and its metabolites influence the proliferation of cholangiocytes (NHC, H69, H69-miR506) and hepatocytes (Hep-G2) in vitro. For this purpose, cells were incubated with 1 nM of DHEA, DHEA-S, E2, adiol or 1 ng/mL of adione and DHT. After 48 h, both DHEA-S and E2 enhanced the proliferation of all the examined cell types (*p* < 0.05; Figure 1A–D). DHEA induced the proliferation of normal human cholangiocytes (NHC and H69), as well as Hep-G2 (*p* < 0.05; Figure 1A,C,D). Additionally, adione significantly increased the proliferation of NHC and Hep-G2 cells (*p* < 0.05; Figure 1C,D), whereas adiol decreased the proliferation of Hep-G2 (*p* < 0.01; Figure 1D). Shorter treatment times of DHEA and its metabolites (24 h) did not change the proliferation of the cell lines (data not shown).

**Figure 2:**
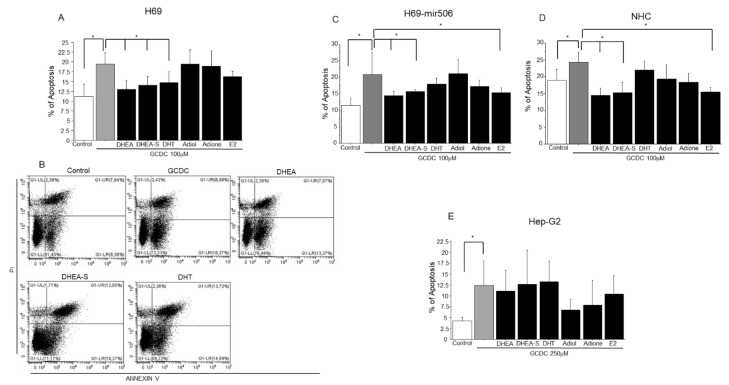
Effect of DHEA and its metabolites on the apoptosis induced by GCDC in cholangiocytes and hepatocytes. H69 (**A**,**B**), H69-mir506 (**C**), NHC (**D**) and Hep-G2 (**E**) cells were incubated with DHEA or its metabolites followed by GCDC. Twenty-four hours following treatment apoptosis was detected using an Annexin V/Propidium iodide (PI) kit. X-axis, Annexin V. Y-axis, PI fluorescence intensities. Representative figures show the population of viable (LL), early apoptotic (LR), late apoptotic (UR) and necrotic (UL) cells. Results are expressed as a percentage of early apoptotic cells from three separate experiments. * Statistically significant difference in comparison to cells treated with GCDC (* *p* < 0.05).

Given that an impaired cholangiocyte response to estrogen characterizes the terminal ductopenic stage of disease (where apoptosis of cholangiocytes is enhanced), the involvement of DHEA and its metabolites in protecting against apoptosis, induced by bile acid (GCDC), was examined. Cells were incubated with DHEA, DHEA-S, E2, adiol, adione or DHT, followed by GCDC incubation (100 μM for NHC, H69 and H69-miR506 cells or 250 μM for Hep-G2 cells) for 24 h. Flow cytometry analysis revealed that DHEA and DHEA-S reduced apoptosis in NHC, H69 and H69-miR506 cells (*p* < 0.05; Figure 2A–D). In addition, DHT diminished apoptosis in H69 cells (*p* < 0.05; Figure 2A). Furthermore, E2 significantly reduced GCDC-induced apoptosis in NHC and H69-miR506 cells (*p* < 0.05; Figure 2C,D). DHEA and its metabolites did not affect GCDC-induced apoptosis in Hep-G2 cells (Figure 2E).

### 3.2. Role of Estrogen and Androgen Receptors in Cholangiocyte and Hepatocyte Apoptosis Induced by GCDC

**Figure 3:**
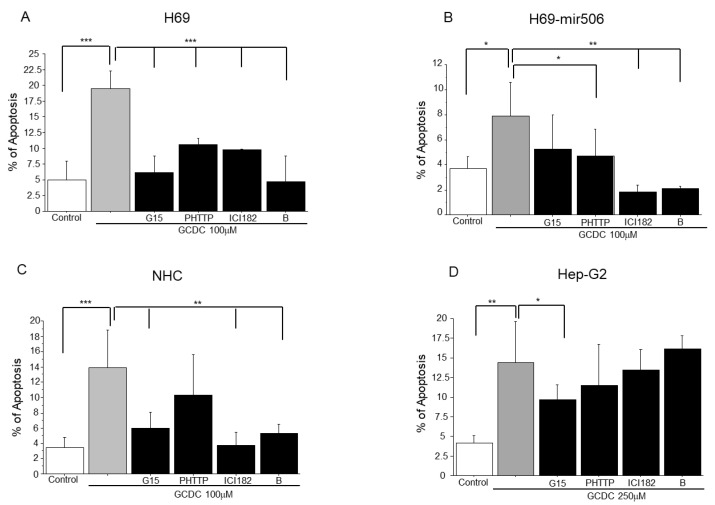
Involvement of estrogen and androgen receptors in the apoptosis induced by GCDC in cholangiocytes (H69, H69-miR506, NHC) and hepatocytes (Hep-G2). Cells (**A**–**D**) were incubated with estrogen (GPER, ER-α, and ER-β) or androgen receptor inhibitors i.e., G15 (12 nM); ICI 182,780 (2 nM); PHTTP (10 nM), and bicalutamide (**B**) (10 nM), followed by GCDC treatment. After 24 h, cells were harvested by scraping and then incubated with Annexin V-FITC (1 ng/mL) and propidium iodide (5 ng/mL) for 30 min in the dark. They were analyzed by CytoFLEX LX flow cytometry, * *p* < 0.05; ** *p* < 0.01, *** *p* < 0.001.

To identify which estrogen receptor was involved in GCDC-induced apoptosis, estrogen or androgen receptor inhibitors—G15 for GPER, ICI 182,780 for ER-α, and PHTTP for ER-β, and bicalutamide for androgen receptors—were used. Cholangiocytes and hepatocytes were incubated with the above-mentioned inhibitors, followed by 100 μM (NHC, H69 and H69-miR506 cells) or 250 μM (Hep-G2 cells) of GCDC. Incubation was prolonged for 24 h. Flow cytometry analysis showed that ICI 182,780 and bicalutamide significantly reduced apoptosis in H69 (*p* < 0.001; Figure 3A), H69-miR506 (*p* < 0.01; Figure 3B) and NHC (*p* < 0.01; Figure 3C) cells, suggesting that ER-α and androgen receptors were involved in bile acid—and GCDC-driven apoptosis in those cells. Furthermore, the ER-β inhibitor and PHTTP reduced apoptosis in both H69 (*p* < 0.001; Figure 3A) and H69-miR506 cells (*p* < 0.05; Figure 3B). The GPER inhibitor and G15 diminished apoptosis in H69 (*p* < 0.001; Figure 3A), NHC (*p* < 0.01; Figure 3C) and Hep-G2 (*p* < 0.05; Figure 3D) cells, but did not change the percentage of apoptosis in H69-miR506 cells (Figure 3D).

### 3.3. Involvement of Estrogen (ER-α, ER-β, and GPER) and Androgen Receptors in DHEA Protection against Mitochondrial Oxidative Stress Induced by tBHQ

**Figure 4:**
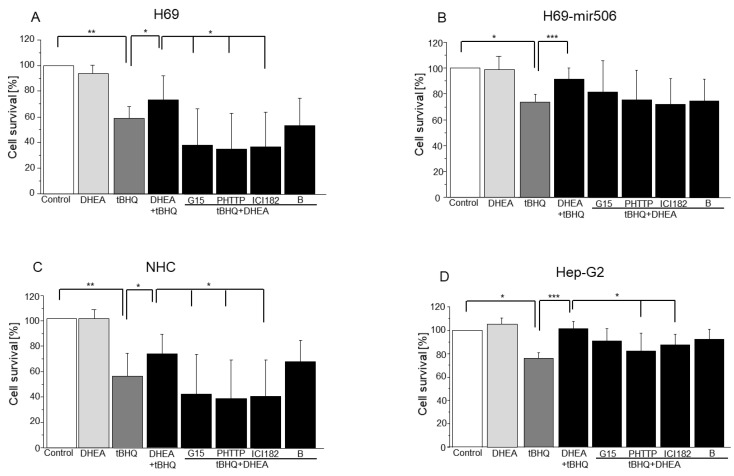
The role of estrogen and androgen receptors in DHEA protection against mitochondrial oxidative stress induced by tBHQ. Cells (**A**–**D**) were incubated with estrogen (GPER, ER-α, and ER-β) or androgen receptor inhibitors i.e., G15 (12 nM); ICI 182,780 (2 nM); PHTTP (10 nM), and bicalutamide (**B**) (10 nM), followed by DHEA and tBHQ co-treatment. To evaluate the viability of examined cells, MTT assays were conducted. Results are presented as mean ± SEM (*n* = 3); * *p* < 0.05, ** *p* < 0.01, *** *p* < 0.001.

The role of estrogens and androgen receptors in the protection of DHEA against oxidative stress was examined. Cells were incubated with inhibitors of estrogen and androgen receptors (G15, 12 nM; ICI 182,780, 2 nM; PHTTP, 10 nM; bicalutamide, 10 nM) for 2 h. Cholangiocytes (H69 and NHC) and hepatocytes (Hep-G2) were then incubated with 100 μM of tBHQ to induce oxidative stress (H69-miR506 cells were incubated with 30 μM of tBHQ) together with 1 nM of DHEA. The MTT assay revealed that ICI 182,780 and PHTTP decreased DHEA protection against oxidative stress in H69, NHC, and Hep-G2 cells (*p* < 0.05; Figure 4A,C,D), while G15 reduced DHEA protection in H69 and NHC cells (*p* < 0.05; Figure 4A,C). In H69-miR506 cells, none of the inhibitors changed the protective effect of DHEA (Figure 4B).

### 3.4. Expression of SULT1E1 and STS in Control and Cirrhotic Human Liver Tissues (PBC and PSC)

As estrogens are metabolized and conjugated primarily in the liver, we investigated the expression of two enzymes (SULT1E1 and STS), which regulate estrogen activities via sulfation/desulfation processes, in cirrhotic (PBC and PSC) and control human liver tissues, using immunohistochemistry and immunoblot.

**Figure 5:**
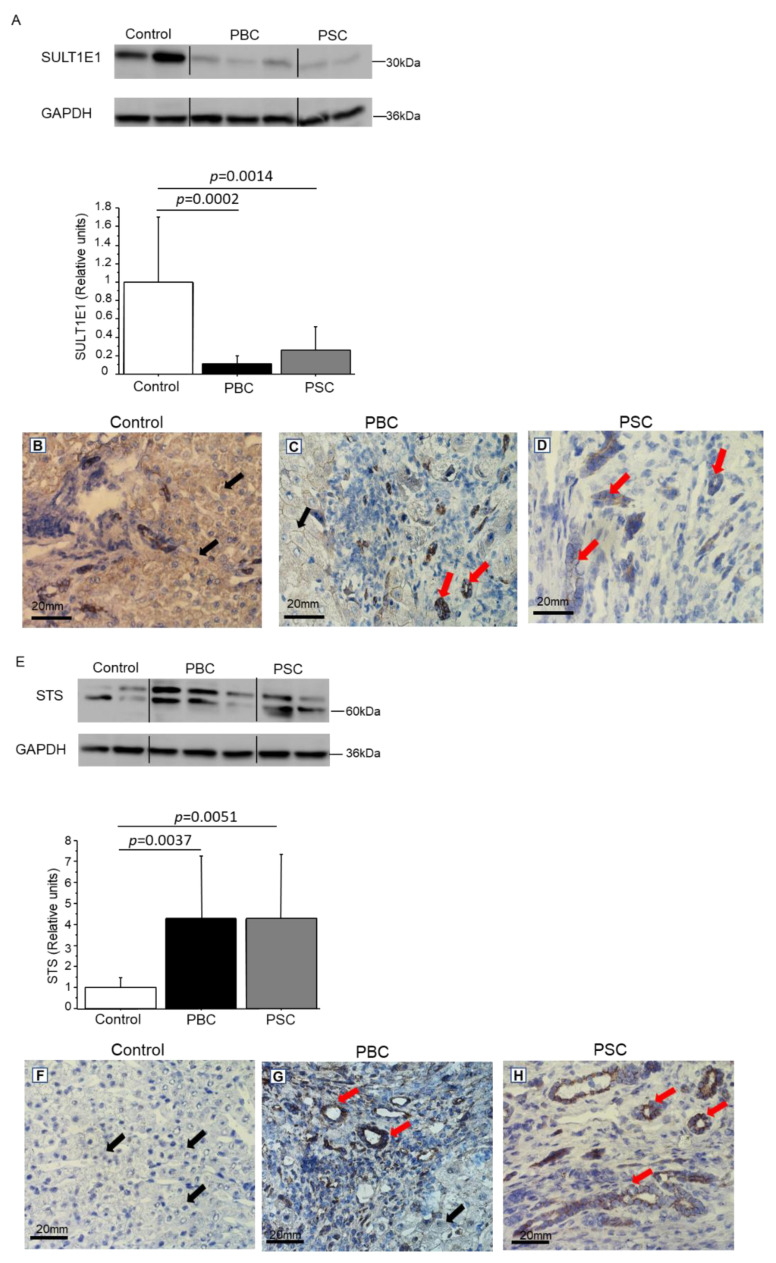
The level and expression of SULT1E1 and STS in cirrhotic (PBC, PSC) and control liver tissues. Western blot analysis revealed lower levels of SULT1E1 (**A**) and higher levels of STS (**E**) in cirrhotic PBC or PSC tissues in comparison to controls. The levels of each protein were normalized to GAPDH as a loading control. Immunohistochemical analysis showed that in control and cirrhotic tissues SULT1E1 was primarily present in cholangiocytes within the bile ducts ((**B**–**D**); red arrows) and in hepatocytes ((**B**,**C**); black arrows). Additionally, in cirrhotic tissues, STS was mainly present in cholangiocytes ((**G**,**H**); red arrows). In control tissues, this protein showed nuclear localization in hepatocytes (**F**). Scale bar—20 μm.

Immunoblot analysis showed that the level of SULT1E1 was reduced in cirrhotic tissue (88% reduction in PBC vs. controls; *p* = 0.0002 and 80% reduction in PSC vs. controls; *p* = 0.0014) (Figure 5A). The level of STS was higher in cirrhotic tissues (4-fold in both PBC and PSC vs. control tissues; *p* = 0.0037 and 0.0051, respectively) (Figure 5E). An immunohistochemical analysis revealed that in cirrhotic tissues, SULT1E1 was mainly present in cholangiocytes within the bile ducts (Figure 5B–D; red arrows) and in hepatocytes (Figure 5B,C; black arrows). Additionally, in cirrhotic tissues, STS was primarily present in cholangiocytes (Figure 5G,H; red arrows), which was in contrast to the control tissues, where the protein was mainly localized in the nuclei of hepatocytes (Figure 5F).

## 4. Discussion

Beyond the well-established role of estrogens in reproductive development, they also play a role in regulating nonreproductive systems, such as immune function [25], metabolism [26,27,28], and growth [29,30].

In this study, we found that DHEA-S and E2 enhanced the proliferation of all investigated liver epithelial cells in vitro. In addition, DHEA increased the proliferation of NHC, H69 and Hep-G2 cells, but not H69-miR506 cells. It has been previously reported that, at micromolar concentrations, DHEA inhibits proliferation, while having a proliferative effect on cells at physiologically relevant, nanomolar concentrations [31,32]. Furthermore, it has been shown that DHEA increases endothelial cell proliferation [33]. E2 and other estrogens are rarely used as proliferative agents, as their presence may lead to the development of cancer. However, E2 can enhance the proliferation of human bone marrow mesenchymal stromal cells, which are considered a possible cell source for regenerative medicine [34].

The results presented here demonstrate that both DHEA and DHEA-S protect NHC, H69 and H69-miR506 cells against apoptosis. Recent evidence suggests an anti-apoptotic effect of DHEA and DHEA-S on neuroendocrine chromaffin cells [35]. Moreover, DHEA and DHEA-S protect PC12 cells against apoptosis via the activation of antiapoptotic Bcl-2 proteins. This pro-survival effect of DHEA and DHEA-S in neuronal PC12 cells is estrogen receptor-independent. Moreover, it involves the activation of the pro-survival transcription factors CREB and NF-κB, upstream effectors of antiapoptotic Bcl-2 protein expression, as well as the pro-survival kinase PKCα/β, a post-translational activator of Bcl-2 [36]. Furthermore, it has been shown that estrogens can protect osteocytes and osteoblasts against apoptosis via activation of the Src/Shc/ERK signaling pathway [37]. DHEA is a metabolic intermediate in the biosynthesis of androgens and estrogens, and its cellular signaling is mediated via a number of different receptors, including nuclear estrogen receptor ER-α and -β, CAR, PXR and PPAR [17]. Recent findings have demonstrated DHEA action via GPER, suggesting there is an alternative means of DHEA action. In addition, ER-α and GPER are the major estrogen receptors expressed in the liver [38]. Our results demonstrate that GPER is involved in apoptosis induced by GCDC in NHC, H69, and Hep-G2 cells, but not in H69-mir506 cells. ER-α and -β and androgen receptors take part in apoptosis induced in cholangiocytes (NHC, H69, and H69-miR506), but not in hepatocytes (Hep-G2). Recently, it has been found that the ER-α-mediated signaling pathway can play multiple roles in the bile duct, which stimulates human intrahepatic biliary epithelial cell proliferation [1].

Mitochondria are important targets of estrogen action [39,40]. The cross-talk between the cell nucleus and mitochondria appears to control estrogen-induced signaling involved in the apoptosis, proliferation, and differentiation of both normal and malignant cells [41,42,43,44,45,46]. Furthermore, mitochondria consume 85% of the oxygen used by the cell, and the mitochondrial electron transport chain generates a substantial amount of intracellular ROS [41]. Given that oxidative stress (OS) could be one of the causes of PBC [23], we investigated whether DHEA could protect against OS induced by tBHQ, and which estrogen or androgen receptors would be involved in this process. In normal cholangiocytes (H69 and NHC cells) DHEA protection against tBHQ was reduced by G15, ICI 182 and PHTTP, which suggests involvement of GPER, ER-α, and androgen receptors. In hepatocytes, GPER and ER-β did not play a role in this protection. DHEA could protect H69-miR506 cells against OS induced by tBHQ, but none of the tested receptors were involved in this process, suggesting that in these cells, which are characterized by a higher level of OS [20], the direct action of DHEA should be considered (and has been shown in other cell types) [47]. It is worth mentioning that DHEA has been identified as an important antioxidant signal that protects adult spinal cord oligodendrocyte precursor cells [32].

SULTs are widely expressed in metabolically active or hormonally responsive tissues, including the liver. SULT1E1 (estrogen sulfotransferase) is best known for its function in sulfo-conjugation and the deactivation of estrogens and plays an important role in human livers [48,49]. Sulfonated estrogens fail to bind to estrogen receptors and, thus, lose their hormonal activities [50]. Our results clearly show that estrogen sulfation is reduced in PBC livers due to the lower expression of SULT1E1 and higher expression of STS, suggesting that estrogen activity is not affected in PBC patients. For the suppression of SULT1E1, it was also reported that cholestasis-induced farnesoid X receptor activation can lead to a reduction in SULT1E1 in cirrhotic liver tissues and, therefore, impede hepatic deactivation of estrogens in PBC [51]. However, STS is a key enzyme that catalyzes the conversion of inactive estrogen sulfates to active estrogen. Estrogens are known for their anti-inflammatory activities [52] and may provide a benefit in regard to inhibiting the progression of chronic inflammatory liver diseases. Moreover, the human STS gene is induced by inflammatory stimuli (LPS and TNF-α) in primary human hepatocytes or human hepatic cell lines. In addition, it has been reported that human STS is a novel NF-κB target gene [53]. In the early stages of liver disease, the activation of NF-κB helps to fight infection and prevent hepatocyte death by inducing anti-apoptotic genes. Moreover, the hepatic expression of STS has been induced in patients with chronic inflammatory liver diseases and was accompanied by an increase in circulating estrogen levels [54]. The regeneration of active DHEA occurs in tissues via the action of steroid sulfatase, which is an important biological function of adipose tissue in postmenopausal women, for whom the major source of estradiol comes from adrenal DHEA-S conversion to estrogens in fat tissue [55].

This study has some limitations. First of all, levels of sulphated estrogens have not been analyzed in cholestatic liver tissues. Additionally, levels of SULT1E1 and STS were not measured in non-cholestatic cirrhotic tissues. That would be of help in better characterization of the specificity of our findings, in the context of advanced cholestasis. We have shown that DHEA expresses its inhibitory effect on oxidative stress in H69 cells via GPER, ER-α and ER-β. However, these receptors were not involved in protection against oxidative stress in H69-miR506 cells. Thus, this study lacks an explanation on the underlying mechanisms of DHEA-mediated protection against oxidative stress in H69-miR506 cells.

Efforts to understand why postmenopausal women predominantly suffer from PBC may lead to the discovery that estrogens regulate a number of steps in the development of PBC. A deeper understanding of the mechanisms of estrogen signaling pathways will likely yield specific targets, governing estrogen’s effect on PBC. Our results showed that, rather than estrogens, it is their precursor DHEA, and its sulfate metabolite DHEA-S, that are involved in sustaining proliferation and depressing apoptosis in cholangiocytes. We believe that there is room for a properly designed clinical study, aimed at the effect of DHEA, on various clinical and biochemical aspects of chronic cholestatic liver conditions. In fact, the beneficial effect of supplementation with DHEA on patients’ health-related quality of life has already been shown [56], but this area definitely requires more thorough studies, which would provide more data on the effect of DHEA in patients with chronic cholestasis.

## Figures and Tables

**Table 1:** Baseline clinical characteristics of analyzed PBC and PSC patients (mean, range values).

Parameters	PBC (*n* = 10)	PSC (*n* = 10)
Age (years)	57 (45–69)	46 (17–62)
Gender (M/F)	(1/9)	(7/3)
AST (IU/L; normal: 5–35)	204 (78–652)	199 (108–510)
ALP (IU/L; normal: 40–120)	639 (234–1373)	548 (171–984)
PLT (10^3^ μl; normal: 150–400)	124 (63–274)	160 (56–321)
Bilirubin (μmol/L; normal: 3.4–20.6)	113 (34–192)	135 (36–316)
Albumin (g/dL; normal: 3.6–4.6)	2.6 (1.7–3.1)	2.2 (1.4–2.9)
INR (normal: 0.8–1.2)	1.3 (1.1–1.7)	1.3 (1.0–1.5)

## Data Availability

The data presented in this study are available upon reasonable request.

## Supplementary Materials

The following supporting information can be downloaded at: https://www.mdpi.com/article/10.3390/cells11061038/s1, Figure S1: Dose-dependent effect of DHEA on cells survival (MTT assay). Results are presented as a mean ± SEM (*n* = 3); * *p* < 0.0 5, ** *p* < 0.01; Figure S2: Time-line scheme of experiments.

## Abbreviations

ER-α, estrogen receptor alpha; ER-β, estrogen receptor beta; CYP19, aromatase cytochrome P450; PXR, pregnane X receptor; CAR, androstan receptor; GPER, G-protein-coupled estrogen receptor; GCDC, glyco-chenodeoxycholic acid; DHEA, dehydroepiandrosterone, E2, 17β-estradiol; E1, estrone; DHT, 5-dihydrotestosterone; InsP3R3, Inositol 1, 4, 5-trisphosphate Receptor Type 3; PAGE, polyacrylamide gel electrophoresis; PBS, phosphate buffer saline; PBC, Primary Biliary Cholangitis; PSC, Primary Sclerosis Cholangitis; FITC, Fluorescein isothiocyanate; SDS, sodium dodecyl sulfate; tBHQ, tert-butylohydrochinon; MTT, 3-(4,5-dimethylthiazol-2-yl)-2,5-diphenyltetrazolium bromide; STS, steroid sulfatase; SULT1E1, sulfotransferase 1E1.

## References

[1] Alvaro D., Gigliozzi A., Attili A.F. (2000). Regulation and deregulation of cholangiocyte proliferation. J. Hepatol..

[2] Alvaro D., Invernizzi P., Onori P., Franchitto A., De Santis A., Crosignani A., Sferra R., Ginanni-Corradini S., Mancino M.G., Maggioni M. (2004). Estrogen receptors in cholangiocytes and the progression of primary biliary cirrhosis. J. Hepatol..

[3] Palmisano B.T., Zhu L., Stafford J.M. (2017). Role of Estrogens in the Regulation of Liver Lipid Metabolism. Adv. Exp. Med. Biol..

[4] Teng Y., Radde B.N., Litchfield L.M., Ivanova M.M., Prough R.A., Clark B.J., Doll M.A., Hein D.W., Klinge C.M. (2015). Dehydroepiandrosterone Activation of G-protein-coupled Estrogen Receptor Rapidly Stimulates MicroRNA-21 Transcription in Human Hepatocellular Carcinoma Cells. J. Biol. Chem..

[5] Van Erpecum K.J., Janssens A.R., Kreuning J., Ruiter D.J., Kroon H.M., Grond A.J. (1988). Generalized peliosis hepatis and cirrhosis after long-term use of oral contraceptives. Am. J. Gastroenterol..

[6] Olsson H.L., Ingvar C., Bladstrom A. (2003). Hormone replacement therapy containing progestins and given continuously increases breast carcinoma risk in Sweden. Cancer.

[7] Chen J., Robertson G., Field J., Liddle C., Farrell G.C. (1998). Effects of bile duct ligation on hepatic expression of female-specific CYP2C12 in male and female rats. Hepatology.

[8] Milkiewicz P., Roma M.G., Cardenas R., Mills C.O., Elias E., Coleman R. (2001). Effect of tauroursodeoxycholate and S-adenosyl-L-methionine on 17beta-estradiol glucuronide-induced cholestasis. J. Hepatol..

[9] Milkiewicz P., Roma M.G., Elias E., Coleman R. (2002). Pathobiology and experimental therapeutics in hepatocellular cholestasis: Lessons from the hepatocyte couplet model. Clin. Sci..

[10] Alvaro D., Mancino M.G., Onori P., Franchitto A., Alpini G., Francis H., Glaser S., Gaudio E. (2006). Estrogens and the pathophysiology of the biliary tree. World J. Gastroenterol..

[11] Alvaro D., Alpini G., Onori P., Franchitto A., Glaser S.S., Le Sage G., Folli F., Attili A.F., Gaudio E. (2002). Alfa and beta estrogen receptors and the biliary tree. Mol. Cell Endocrinol..

[12] Secky L., Svoboda M., Klameth L., Bajna E., Hamilton G., Zeillinger R., Jager W., Thalhammer T. (2013). The sulfatase pathway for estrogen formation: Targets for the treatment and diagnosis of hormone-associated tumors. J. Drug Deliv..

[13] Yi M., Negishi M., Lee S.J. (2021). Estrogen Sulfotransferase (SULT1E1): Its Molecular Regulation, Polymorphisms, and Clinical Perspectives. J. Pers. Med..

[14] Alnouti Y. (2009). Bile Acid sulfation: A pathway of bile acid elimination and detoxification. Toxicol. Sci..

[15] Labrie F. (2010). DHEA, important source of sex steroids in men and even more in women. Prog. Brain Res..

[16] Ahboucha S., Pomier-Layrargues G., Vincent C., Hassoun Z., Tamaz R., Baker G., Butterworth R.F. (2008). Reduced plasma dehydroepiandrosterone sulfate levels are significantly correlated with fatigue severity in patients with primary biliary cirrhosis. Neurochem. Int..

[17] Prough R.A., Clark B.J., Klinge C.M. (2016). Novel mechanisms for DHEA action. J. Mol. Endocrinol..

[18] Webb S.J., Geoghegan T.E., Prough R.A., Michael Miller K.K. (2006). The biological actions of dehydroepiandrosterone involves multiple receptors. Drug Metab. Rev..

[19] Liu D., Iruthayanathan M., Homan L.L., Wang Y., Yang L., Wang Y., Dillon J.S. (2008). Dehydroepiandrosterone stimulates endothelial proliferation and angiogenesis through extracellular signal-regulated kinase 1/2-mediated mechanisms. Endocrinology.

[20] Erice O., Munoz-Garrido P., Vaquero J., Perugorria M.J., Fernandez-Barrena M.G., Saez E., Santos-Laso A., Arbelaiz A., Jimenez-Aguero R., Fernandez-Irigoyen J. (2018). MicroRNA-506 promotes primary biliary cholangitis-like features in cholangiocytes and immune activation. Hepatology.

[21] Kilanczyk E., Banales J.M., Wunsch E., Barbier O., Avila M.A., Mato J.M., Milkiewicz M., Milkiewicz P. (2020). S-adenosyl-L-methionine (SAMe) halts the autoimmune response in patients with primary biliary cholangitis (PBC) via antioxidant and S-glutathionylation processes in cholangiocytes. Biochim. Biophys. Acta Mol. Basis Dis..

[22] Banales J.M., Saez E., Uriz M., Sarvide S., Urribarri A.D., Splinter P., Tietz Bogert P.S., Bujanda L., Prieto J., Medina J.F. (2012). Up-regulation of microRNA 506 leads to decreased Cl-/HCO3- anion exchanger 2 expression in biliary epithelium of patients with primary biliary cirrhosis. Hepatology.

[23] Wasik U., Milkiewicz M., Kempinska-Podhorodecka A., Milkiewicz P. (2017). Protection against oxidative stress mediated by the Nrf2/Keap1 axis is impaired in Primary Biliary Cholangitis. Sci. Rep..

[24] Orentreich N., Brind J.L., Rizer R.L., Vogelman J.H. (1984). Age changes and sex differences in serum dehydroepiandrosterone sulfate concentrations throughout adulthood. J. Clin. Endocrinol. Metab..

[25] Khan D., Ansar Ahmed S. (2015). The Immune System Is a Natural Target for Estrogen Action: Opposing Effects of Estrogen in Two Prototypical Autoimmune Diseases. Front. Immunol..

[26] Klair J.S., Yang J.D., Abdelmalek M.F., Guy C.D., Gill R.M., Yates K., Unalp-Arida A., Lavine J.E., Clark J.M., Diehl A.M. (2016). A longer duration of estrogen deficiency increases fibrosis risk among postmenopausal women with nonalcoholic fatty liver disease. Hepatology.

[27] Hussain Y., Ding Q., Connelly P.W., Brunt J.H., Ban M.R., McIntyre A.D., Huff M.W., Gros R., Hegele R.A., Feldman R.D. (2015). G-protein estrogen receptor as a regulator of low-density lipoprotein cholesterol metabolism: Cellular and population genetic studies. Arterioscler. Thromb. Vasc. Biol..

[28] Zhang Z.C., Liu Y., Xiao L.L., Li S.F., Jiang J.H., Zhao Y., Qian S.W., Tang Q.Q., Li X. (2015). Upregulation of miR-125b by estrogen protects against non-alcoholic fatty liver in female mice. J. Hepatol..

[29] Leung K.C., Johannsson G., Leong G.M., Ho K.K. (2004). Estrogen regulation of growth hormone action. Endocr. Rev..

[30] Avtanski D., Novaira H.J., Wu S., Romero C.J., Kineman R., Luque R.M., Wondisford F., Radovick S. (2014). Both estrogen receptor alpha and beta stimulate pituitary GH gene expression. Mol. Endocrinol..

[31] Lopez-Marure R., Contreras P.G., Dillon J.S. (2011). Effects of dehydroepiandrosterone on proliferation, migration, and death of breast cancer cells. Eur. J. Pharmacol..

[32] Kilanczyk E., Saraswat Ohri S., Whittemore S.R., Hetman M. (2016). Antioxidant Protection of NADPH-Depleted Oligodendrocyte Precursor Cells Is Dependent on Supply of Reduced Glutathione. ASN Neuro.

[33] Williams M.R., Dawood T., Ling S., Dai A., Lew R., Myles K., Funder J.W., Sudhir K., Komesaroff P.A. (2004). Dehydroepiandrosterone increases endothelial cell proliferation in vitro and improves endothelial function in vivo by mechanisms independent of androgen and estrogen receptors. J. Clin. Endocrinol. Metab..

[34] Hong L., Zhang G., Sultana H., Yu Y., Wei Z. (2011). The effects of 17-beta estradiol on enhancing proliferation of human bone marrow mesenchymal stromal cells in vitro. Stem Cells Dev..

[35] Charalampopoulos I., Tsatsanis C., Dermitzaki E., Alexaki V.I., Castanas E., Margioris A.N., Gravanis A. (2004). Dehydroepiandrosterone and allopregnanolone protect sympathoadrenal medulla cells against apoptosis via antiapoptotic Bcl-2 proteins. Proc. Natl. Acad. Sci. USA.

[36] Charalampopoulos I., Alexaki V.I., Tsatsanis C., Minas V., Dermitzaki E., Lasaridis I., Vardouli L., Stournaras C., Margioris A.N., Castanas E. (2006). Neurosteroids as endogenous inhibitors of neuronal cell apoptosis in aging. Ann. N. Y. Acad. Sci..

[37] Kousteni S., Han L., Chen J.R., Almeida M., Plotkin L.I., Bellido T., Manolagas S.C. (2003). Kinase-mediated regulation of common transcription factors accounts for the bone-protective effects of sex steroids. J. Clin. Investig..

[38] Prossnitz E.R., Barton M. (2014). Estrogen biology: New insights into GPER function and clinical opportunities. Mol. Cell Endocrinol..

[39] Klinge C.M. (2017). Estrogens regulate life and death in mitochondria. J. Bioenerg. Biomembr..

[40] Klinge C.M. (2008). Estrogenic control of mitochondrial function and biogenesis. J. Cell Biochem..

[41] Felty Q., Roy D. (2005). Estrogen, mitochondria, and growth of cancer and non-cancer cells. J. Carcinog..

[42] Lewis-Wambi J.S., Jordan V.C. (2009). Estrogen regulation of apoptosis: How can one hormone stimulate and inhibit?. Breast Cancer Res..

[43] Belcredito S., Brusadelli A., Maggi A. (2000). Estrogens, apoptosis and cells of neural origin. J. Neurocytol..

[44] Nilsen J., Chen S., Irwin R.W., Iwamoto S., Brinton R.D. (2006). Estrogen protects neuronal cells from amyloid beta-induced apoptosis via regulation of mitochondrial proteins and function. BMC Neurosci..

[45] Yang S.D., Ma L., Gu T.X., Ding W.Y., Zhang F., Shen Y., Zhang Y.Z., Yang D.L., Zhang D., Sun Y.P. (2014). 17beta-Estradiol protects against apoptosis induced by levofloxacin in rat nucleus pulposus cells by upregulating integrin alpha2beta1. Apoptosis.

[46] Spyridopoulos I., Sullivan A.B., Kearney M., Isner J.M., Losordo D.W. (1997). Estrogen-receptor-mediated inhibition of human endothelial cell apoptosis. Estradiol as a survival factor. Circulation.

[47] Traish A.M., Kang H.P., Saad F., Guay A.T. (2011). Dehydroepiandrosterone (DHEA)--a precursor steroid or an active hormone in human physiology. J. Sex. Med..

[48] Xie Y., Xie W. (2020). The Role of Sulfotransferases in Liver Diseases. Drug Metab. Dispos..

[49] Barbosa A.C.S., Feng Y., Yu C., Huang M., Xie W. (2019). Estrogen sulfotransferase in the metabolism of estrogenic drugs and in the pathogenesis of diseases. Expert Opin. Drug Metab. Toxicol..

[50] Song W.C. (2001). Biochemistry and reproductive endocrinology of estrogen sulfotransferase. Ann. N. Y. Acad. Sci..

[51] Liu X., Xue R., Yang C., Gu J., Chen S., Zhang S. (2018). Cholestasis-induced bile acid elevates estrogen level via farnesoid X receptor-mediated suppression of the estrogen sulfotransferase SULT1E1. J. Biol. Chem..

[52] Straub R.H. (2007). The complex role of estrogens in inflammation. Endocr. Rev..

[53] Ding W.X., Yin X.M. (2004). Dissection of the multiple mechanisms of TNF-alpha-induced apoptosis in liver injury. J. Cell Mol. Med..

[54] Jiang M., Klein M., Zanger U.M., Mohammad M.K., Cave M.C., Gaikwad N.W., Dias N.J., Selcer K.W., Guo Y., He J. (2016). Inflammatory regulation of steroid sulfatase: A novel mechanism to control estrogen homeostasis and inflammation in chronic liver disease. J. Hepatol..

[55] Labrie F., Belanger A., Belanger P., Berube R., Martel C., Cusan L., Gomez J., Candas B., Chaussade V., Castiel I. (2007). Metabolism of DHEA in postmenopausal women following percutaneous administration. J. Steroid. Biochem. Mol. Biol..

[56] Wronka K.M., Wunsch E., Kozlowska-Petriczko K., Wojcicki M., Kruk B., Milkiewicz P. (2021). Dehydroepiandrosterone sulfate indicates decreased sulfation capacity and impaired quality of life in patients with primary sclerosing cholangitis. Pol. Arch. Intern. Med..

