# Supplementary material for: DHEA Protects Human Cholangiocytes and Hepatocytes against Apoptosis and Oxidative Stress
Source: Cells. 2022 Mar 18;11(6):1038. doi: 10.3390/cells11061038 (PMC8947473; doi:10.3390/cells11061038)
Supplement: Supplementary file 1 [file cells-11-01038-s001.zip › cells-1634429-supplementary.pdf]

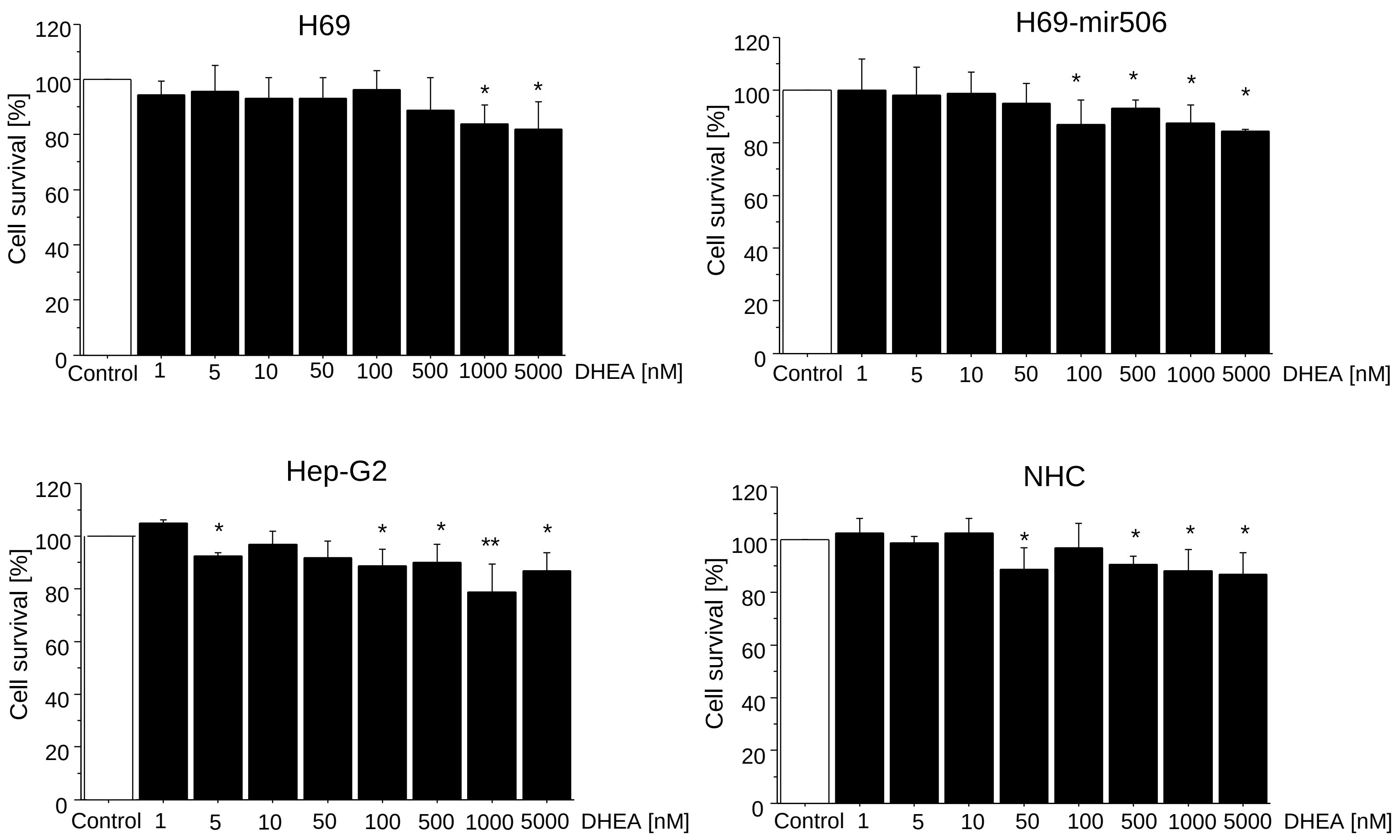

Figure S1. Dose - dependent effect of DHEA on cells survival (MTT assay). Results are presented as a mean±SEM (n=3); *p<0.05; **p<0.01.

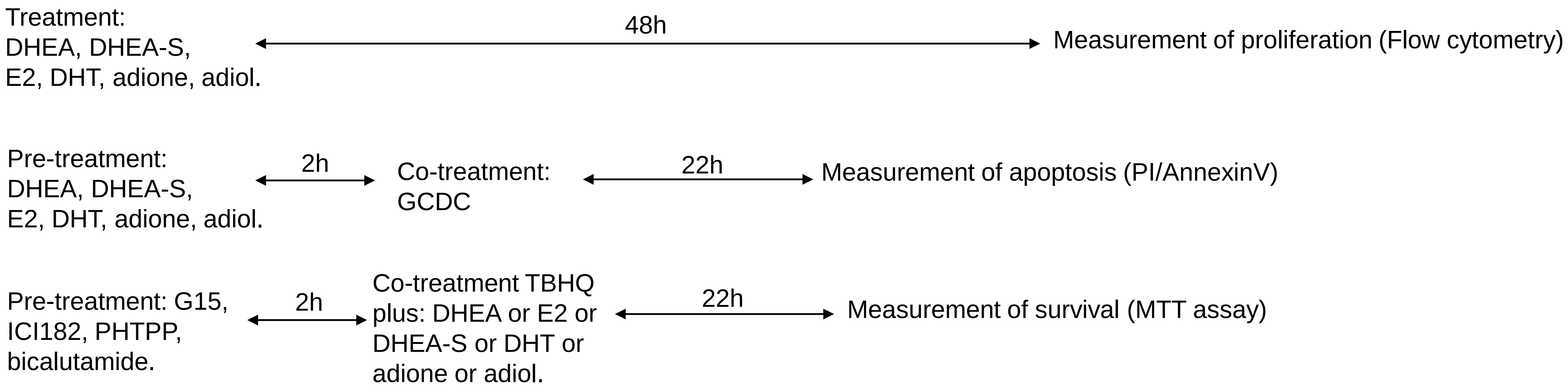

Figure S2. Time-line scheme of experiments
